# Dietary Inflammatory Index and Cardiometabolic Risk in Ecuadorian Women

**DOI:** 10.3390/nu13082640

**Published:** 2021-07-30

**Authors:** Yankun Wang, Rodrigo X. Armijos, Pengcheng Xun, Mary Margaret Weigel

**Affiliations:** 1 Department of Environmental & Occupational Health, Indiana University-Bloomington School of Public Health, Bloomington, IN 47405, USA; wangyank@iu.edu (Y.W.); rarmijos@iu.edu (R.X.A.); 2 Global Environmental Health Research Laboratory, Indiana University-Bloomington School of Public Health, Bloomington, IN 47405, USA; 3 Center for Latin American & Caribbean Studies, Indiana University-Bloomington, Bloomington, IN 47405, USA; 4 Department of Epidemiology and Biostatistics, Indiana University-Bloomington School of Public Health, Bloomington, IN 47405, USA; pxun@indiana.edu; 5 Atara Biotherapeutics, Thousand Oaks, CA 91320, USA

**Keywords:** dietary inflammation index, metabolic syndrome, cardiometabolic risk factors, Ecuador

## Abstract

Low-grade systemic inflammation is implicated in metabolic syndrome (MetS) and cardiometabolic diseases. Diet is hypothesized to be an important low-grade inflammation modifier. However, few studies have examined the association of dietary inflammation with MetS and cardiometabolic risk in Latin American populations and their findings are inconsistent. Our cross-sectional study examined the association of dietary inflammatory potential with MetS and cardiometabolic risk components in 276 urban Ecuadorian women. Dietary inflammation was evaluated using an energy-adjusted Dietary Inflammatory Index (E-DII), divided into quartiles (Q). E-DII scores ranged from −4.89 (most anti-inflammatory) to 4.45 (most pro-inflammatory). Participants in the most pro-inflammatory (Q4) compared to the least inflammatory E-DII quartile (Q1) had a 4.4 increased adjusted odds for MetS (95% C.I. = 2.0, 9.63; *p* < 0.001). Every one-unit increase in E-DII was associated with a 1.4 increase in MetS (95% CI = 1.22, 1.52; *p* < 0.001). In other adjusted models, the most pro-inflammatory E-DII quartile (Q4) was positively associated with total blood cholesterol and triglycerides (*p* < 0.001), LDL-c (*p* = 0.007), diastolic blood pressure (*p*< 0.002), mean arterial pressure (*p* < 0.006), waist circumference (*p* < 0.008), and Framingham risk score (*p* < 0.001). However, the previously identified associations with pulse wave velocity and BMI were no longer evident in the models. These findings suggest that more pro-inflammatory diets may contribute to poorer cardiometabolic health. Promoting healthier diets with a lower inflammatory potential may help to prevent or slow development of cardiometabolic disorders.

## 1. Introduction

Cardiometabolic disorders, such as type 2 diabetes (T2D), cardiovascular disease (CVD), stroke, and fatty liver disease, pose a major and growing global public health challenge. These are characterized by the presence of a common group of metabolic risk factors including obesity, impaired glucose regulation, and dyslipidemia [1]. Population rates of cardiometabolic disease and metabolic syndrome (MetS), the cluster of risk factors that raises the risk for these, are rapidly increasing especially in low- and middle-income countries (LMICs) undergoing nutritional and epidemiologic transition [2] including those in Latin America [3].

Chronic low-grade inflammation is an important feature of cardiometabolic disease. It is caused by exposures that promote excessive production of oxygen-reactive species (ROS), generation of pro-inflammatory cytokines, adipokines, and other pro-inflammatory responses [4,5]. Over time, this can lead to metabolic derangements, metabolic syndrome, and cardiometabolic diseases. C-reactive protein (CRP) and interleukin-6 (IL-6) are pro-inflammatory biomarkers linked to cardiometabolic disease risk [4,6,7,8]. In contrast, anti-inflammatory biomarkers, such as adiponectin, interleukin-4 (IL-4), and interleukin-10 (IL-10), are reported as associated with lower risk for T2D, CVD, and other cardiometabolic conditions [8,9,10,11].

Diet appears to be an important modifiable modulator of low-grade systemic inflammation. Published studies from diverse populations suggest that the frequent consumption of energy-dense, micronutrient- and fiber-poor diets such as the “Western diet” appears to promote oxidative stress, innate immune system activation, and pro-inflammatory/anti-inflammatory cytokine ratio imbalance, which over time, can lead to chronic low-grade inflammation and cardiometabolic derangements [12].

A growing body of evidence suggests that the frequent consumption of fruits and vegetables, whole grains, nuts and legumes, fish, olive oil, and other minimally processed foods such as is characteristics of the “Mediterranean” and “DASH” (Dietary Approaches to Stop Hypertension) diets are associated with less systemic inflammation and lower cardiometabolic risk [12,13,14,15]. Accumulating evidence suggests that diet type also can influence gut microbiome composition. This is important since pro- and anti-inflammatory metabolites generated by the gut microbiome can pass through the gastrointestinal barrier and enter into the systemic circulation to contribute and/or ameliorate inflammation and cardiometabolic risk [16,17,18].

Compared to single foods or nutrients, overall dietary patterns tend to be more informative about the habitual diet of individuals and effects on nutrition and health. However, knowing an individual’s general diet pattern provides only limited information on its overall inflammatory potential [15]. The Dietary Inflammatory Index was developed to better address the overall inflammatory potential of individual diets [19]. The impact of different food parameters (i.e., individual food items, micro/macro nutrients, and flavonoids) on inflammation from the published literature was analyzed to construct the index. Specifically, the pro-inflammatory or anti-inflammatory effects of food parameters were assessed by the production of specific inflammatory blood biomarkers such as interleukin (IL)-1β, IL-4, IL-6, IL-10, tumor necrosis factor alpha (TNF-α) and/or C-reactive protein (CRP). The index is standardized to a regionally representative world database. To control for the effects of differences among individuals in their total energy intakes, energy-adjusted scores are calculated per 1000 kcal of food consumed [19].

Cohort and cross-sectional studies investigating the association between energy-adjusted Dietary Inflammatory Index E-DII scores with MetS and/or individual cardiometabolic risk biomarkers have reported mixed findings for diverse populations from North America [20,21,22], Europe [23,24,25,26,27,28], Asia and the Middle East [29,30,31,32]. Published studies reporting on association of E-DII scores with MetS and/or cardiometabolic risk components in Latin American adults are limited to three countries, i.e., Brazil [33], Colombia [34] and Mexico [35,36] and their findings also are mixed.

Examination of the dietary inflammation potential of Latin America diets and their relationship with MetS and cardiometabolic risk factors is an important topic of inquiry. While countries in different regions of Latin America have very different diet, two features that most share in common is a declining intake of fresh fruits and vegetables, and whole-grains and an increasing consumption of energy-dense, micronutrient-poor processed/ultra-processed foods and beverages high in saturated and trans-fats, sugars and other refined carbohydrates, red meats, and sodium [37,38]. These dietary trends, along with decreasing physical activity, increasing sedentarism, and other exposures have been linked to rapidly rising rates of obesity, other individual cardiometabolic risk factors, MetS, and cardiometabolic disease morbidity, mortality, and disability in the region, including among adult women [3,37,38].

Ecuador has a high population burden of metabolic and other chronic diseases. In 2019, heart disease, T2D, and cerebrovascular disease ranked among the top five causes of mortality in adults over the age of 30 years [39]. A 2014 national survey found that adults aged <60 years have a high prevalence of overweight/obesity, abdominal obesity, hypertriglyceridemia, low HDL-c cholesterol, hyperglycemia and high blood pressure [40]. Another study identified MetS in nearly one-third [41]. Although no sex-specific differences were identified in the prevalence of MetS, women were more likely than men to show evidence of generalized and abdominal obesity, low HDL-c, and hypertension [41]. The inflammatory potential of Ecuadorian diets has not yet been characterized using the E-DII. However, studies have reported that traditional diets rich in fruits, vegetables, whole grains and other foods rich in micronutrients, phytonutrients, and fiber appear to be on the decline while that of processed/ultra-processed foods and sweetened beverages is rising [42,43].

We conducted a study to characterize the inflammatory potential of the diets of Ecuadorian women using an energy-adjusted E-DII and to investigate the association of E-DII scores with MetS and individual cardiometabolic risk factors including waist circumference, body mass index (BMI), blood glucose, blood lipids (total cholesterol, HDL-c, LDL-c, triglycerides), blood pressure, and pulse wave velocity, an indicator of endothelial dysfunction (arterial stiffness). Our working hypothesis was that compared to women with less inflammatory diets, those whose diets were more pro-inflammatory would show evidence of MetS as well as individual anthropometric (higher BMI and waist circumference), laboratory (elevated blood glucose, total cholesterol, LDL-c, and triglyceride levels, and lower HDL-c), and clinical cardiometabolic risk components (higher blood pressure, greater arterial stiffness).

## 2. Materials and Methods

### 2.1. Study Site and Population

We analyzed data collected during a previous study of maternal-child dyads in Quito, Ecuador. The sampling methods and participant inclusion criteria used in the survey-design study have been previously described in detail [44]. Briefly, 279 primary school children and their mothers or other adult maternal caregivers living in the same home were recruited from local public schools. Maternal-child dyads were selected for potential participation using a computerized random numbers program. The inclusion criteria for mothers included current residence in the same home as the selected child, not have another child participating in the study, not be affected by any condition making it difficult to understand or respond to the interview questions, and not have a history of or show evidence of any major cardiometabolic, autoimmune, or other pro-inflammatory chronic or infectious conditions (e.g., diabetes, hypertension, TB, HIV/AIDS).

Participants provided their written and oral informed consent prior to the start of data collection. The study was conducted following Declaration of Helsinki guidelines. The protocol was approved by the University of Texas at El Paso Institutional Review Board (#604134) and Bioethics Committee (COBI) of the Central University of Ecuador. All study participants received oral and written interpretations of their lab, diet, and health screenings. Those identified with clinically abnormal results were referred to public health system facilities for free follow-up care.

### 2.2. Data Collection

Face-to-face interviews were used to collect data on participant and household sociodemographic characteristics, health history, lifestyle (smoking), and usual dietary intakes. In addition, data were collected on cardiometabolic risk factors including anthropometric, clinical, and laboratory indicators.

#### 2.2.1. Sociodemographic and Lifestyle Characteristics

A structured questionnaire, validated in prior nutritional and health studies in Ecuador was used to collect data on the sociodemographic characteristics of the women participants and their households [42,44]. These included age, birthplace, ethnicity, marital status, education, current employment status, occupation, monthly household income, family size, place and length of residence. They were also asked about their smoking status.

#### 2.2.2. Dietary Intake

We used a semi-quantitative FFQ to evaluate participant dietary intake that was previously developed and validated during a prior study of women living in low-income neighborhoods in the same highland Ecuadorian city (Quito) [43]. The FFQ collected detailed information from the women participants on their usual intake of 133 foods and beverages during the past 12-months. Possible responses on intake frequency for each FFQ item ranged from a low of never or less than once per month to a high of 4–6 times a day. Possible responses for the portion size of these was small, medium, or large.

#### 2.2.3. Dietary Inflammatory Index

In our study, we calculated the E-DII using data collected from participant FFQs. These included 39 of the 45 foods, nutrients, and phytochemicals contained in the original DII [19]. These included carbohydrates, protein, total fat, cholesterol, fiber, saturated fatty acids, monounsaturated fatty acids, polyunsaturated fatty acids, omega-3 fatty acids, omega-6 fatty acids, trans fats, niacin, thiamin, riboflavin, iron, magnesium, zinc, selenium, vitamin A, vitamin B_6_, vitamin B_12_, vitamin C, vitamin D, vitamin E, folic acid, beta carotene, grams of alcohol, caffeine, garlic, onion, black tea, pepper, flavan-3-ol, flavones, flavanols, flavanones, isoflavones, and anthocyanins. Six components included in the original DII (saffron, turmeric, ginger, rosemary, eugenol, thyme) were not analyzed in our study because data on them were not available from the FFQ. The values for the 39 foods and food constituents used in the study were obtained from the USDA Agricultural Research Service Nutrient Data Laboratory databases (Beltsville, MD). We obtained the E-DII by estimating the energy-adjusted intakes of each food item using the FFQ. Each participant’s energy-adjusted dietary inflammatory index (E-DII) was calculated using the updated method published by Shivappa and associates [45]. To control for the influence of energy intake, the E-DII was calculated per 1000 kcals of food consumed.

**Figure 1:**
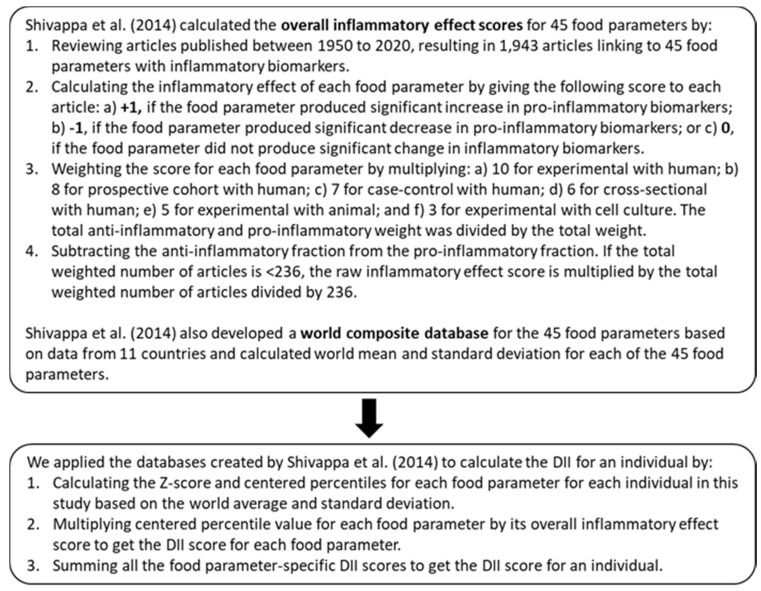
Overview of steps involved in the calculation of the energy-adjusted Dietary Inflammatory Index (E-DII) scores.

Figure 1 shows the steps we followed to calculate the E-DII for an individual. Using this method, lower E-DII scores indicate a more anti-inflammatory diet, and higher scores indicate a more pro-inflammatory diet.

#### 2.2.4. Anthropometric Indicators

Data on participant anthropometric characteristics were collected using published protocols [46]. Participants were weighed to the nearest kilogram on a calibrated electronic scale (Detecto, Webb City, MO, USA) without shoes, coats, or other heavy clothing. The scale was recalibrated after each weighing. The standing height of participants, who were without shoes, hats, or other headwear, was measured using a portable stadiometer (Seca NA, Chino, CA, USA). The weight and height measurements obtained were used to calculate body mass index (BMI) defined as weight (kg)/height (m)^2^. Waist circumference was measured using a semiflexible anthropometric measuring tape (Seca NA, Chino, CA, USA).

#### 2.2.5. Clinical and Laboratory Indicators

Blood pressure (BP) was measured following the American Heart Association recommended protocol [47]. Seated participants rested in a seated position for 15 min before their blood pressures were measured from their right arm. Measurements were made using a calibrated automatic medical sphygmomanometer with an adjustable cuff (Omron Health Care, Lake Forrest, IL, USA). Systolic (SBP) and diastolic blood pressures (DBP) were measured to the nearest 2 mm Hg.

A second measurement was made 10 min later and the average of the two values was recorded. In addition, we calculated mean arterial pressure (MAP), i.e., the average BP over a cardiac cycle, using a published formula: MAP = DBP + 1/3 (SBP − DBP) [48]. In addition to blood perfusion to organs, MAP is used as a surrogate marker for vascular stiffness subsequent to loss of vascular elasticity [49].

Data on pulse wave velocity (PWV), a measure of vascular endothelial dysfunction (arterial stiffening) were collected on all participants by a trained, experienced physician-scientist (RA) using the SphygmoCor XCEL (AtCor Medical, Australia) and following the protocol published by Nordstrand and associates [50].

Capillary blood samples obtained from participant fingertips were used to measure fasting blood glucose and lipids (total cholesterol, LDL-c, HDL-c, triglycerides). Samples were analyzed using the Cholestech Cholestech L-D-X System (Alere, San Diego, CA, USA) following manufacturer specifications. The instrument was calibrated prior to each measurement session.

We used the joint International Diabetes Federation/U.S. National Heart, Lung, and Blood Institute/American Heart Association/World Heart Federation harmonized criteria for MetS [1]. MetS was defined when three or more of the following risk factors were present: waist circumference ≥ 80 cm, elevated fasting blood triglycerides (≥150 mg/dL), low high-density lipoprotein cholesterol (HDL-c) (≤40 mg/dL), elevated fasting blood glucose (≥100 mg/dL), or elevated systolic (≥130 mmHg) and/or diastolic blood pressure (≥85 mmHg).

We calculated the cardiovascular risk of the women participants using the Framingham Risk Score algorithm. This is a gender-specific algorithm that estimates the 10-year cardiovascular risk of an individual [51].

### 2.3. Data Analysis

Three of the women in the study were missing FFQ data so their E-DIIs could not be calculated. For that reason, we did not include them in any of the analyses. The summary statistics for participant and household sociodemographic characteristics were analyzed as proportions or mean ± SD. The E-DII scores were analyzed as both continuous variables and quartiles for the risk indicators of interest in this study. Differences in sociodemographic characteristics by E-DII quartiles were analyzed using students’ independent t-test or one-way analysis of variance (ANOVA), as appropriate, for continuous variables and X^2^ test for categorical variables. Binary logistic regression models were used to examine the unadjusted and adjusted associations of the E-DII quartiles with MetS. We used linear regression to examine the unadjusted and adjusted associations for cardiometabolic risk indicators across E-DII quartiles. We also assessed the association of the E-DII with participant cardiovascular risk using the Framingham Risk Score (FRS) algorithm [51] using linear regression.

The adjusted models controlled for variables previously identified in the literature as potential confounders [45,52,53,54,55]. These included age, sex, ethnicity, education, smoking, poverty index, physical activity. We also adjusted for BMI except in the BMI and waist circumference models. All risk indicators in this study were log-transformed before linear regression analysis due to their non-normal distributions. The study data were analyzed using SAS statistical software (version 9.4, Cary, NC, USA). In this study, *p* values < 0.05 (2-tailed test) were considered as statistically significant.

## 3. Results

Table 1 summarizes the sociodemographic, fasting blood glucose and lipid parameters, and other characteristics of the 276 women participants and their households. The average age of participants was 36 years, and the majority were from the mestizo ethnic majority group. Most participants had a primary school education, and most were employed outside the home. The *per capita* income of the participants averaged $125 US dollars/month. None of the women participants reported being cigarette smokers. In our study, E-DII scores ranged between −4.89 and 4.45. We transformed the continuous scores into quartiles (Q): Q1 was the most anti-inflammatory (−4.89 to −3.43), Q2 was anti-inflammatory (−3.42 to −1.63), Q3 was pro-inflammatory (−1.62 to 1.16), and Q4 was the most pro-inflammatory (1.17 to 4.45).

As Table 1 shows, no statistically significant differences were identified between E-DII quartiles and participant age, ethnicity, income, or the other sociodemographic attributes measured in the study except for marital status. Women in the two most pro-inflammatory quartiles had slightly higher average blood pressures although the difference was too small to be clinically meaningful. Those with the most pro-inflammatory diets (Q4) had significantly greater average blood values of total cholesterol, triglycerides, LDL-c, and greater waist circumference but not HDL-c or blood glucose.

Table 2 shows the average daily intakes of the food parameters used in calculating the E-DII quartiles. Women participants in the higher E-DII quartiles tended to have higher average intakes of total fat and carbohydrates reported in the literature to promote inflammation and generally lower intakes of most micronutrients, polyphenols and spices reported as having more anti-inflammatory properties. In contrast, participants in the most anti-inflammatory E-DII quartiles generally had higher average dietary intakes of fiber, n-fatty acids, and most micronutrients and polyphenols linked in the literature with inflammatory properties. However, their reported average intakes of some constituents such as cholesterol and saturated fats was somewhat higher on average, perhaps due to higher intakes of eggs, fish, and other animal products that also tend to be rich in food constituents such as n-fatty acids and micronutrients (e.g., vitamin A, zinc, iron).

Nearly one-quarter (24.6%) of the participants met the criteria for MetS. Table 3 displays the findings from the logistic regression analyses examining the association of E-DII score quartiles with MetS. E-DII scores were significantly associated with the odds of MetS in both the unadjusted and covariate-adjusted models and this relationship appeared to be dose-response. The table shows that in Model 1, subjects with the most pro-inflammatory diets (Q4) were approximately 4.5 times more likely to meet the criteria for MetS compared to those with the most anti-inflammatory diet (Q1). After adjusting covariates in Model 2, the odds of MetS among Q4 participants was increased 4.4 times over that of those in Q1. Table 2 also indicates that for each one-unit increase in E-DII score, the odds of MetS rose by approximately 1.4 times in both the unadjusted and adjusted analyses.

Table 4 displays the findings from models examining the association between E-DII score quartile with cardiometabolic risk indicators. In the unadjusted model (Model 1), Q4 E-DII score quartiles were positively associated with total blood cholesterol, LDL-C, and triglyceride levels. It was also positively associated with pulse wave velocity, diastolic blood pressure, mean arterial pressure, BMI and waist circumference but not blood glucose, HDL-c, or systolic blood pressure.

The covariate-adjusted models (Model 2), showed similar results for the positive associations of E-DII Q4 scores with total blood cholesterol, LDL-C, and triglyceride levels. It was also positively associated with diastolic blood pressure, mean arterial pressure, and waist circumference. However, the previously identified associations with pulse wave velocity and BMI were no longer evident in the models.

Table 5 shows the findings from the analysis examining the association of E-DII quartiles with the Framingham Risk Scores (FRS) of the women participants. Those in the most anti-inflammatory E-DII quartile (Q1) had the lowest average FRS scores and those with the those in the most pro-inflammatory quartile (Q4) had the highest. The results identified a linear association between the E-DII and their FRS (linear rend *p* < 0.01).

## 4. Discussion

To the best of knowledge, this is the first study to examine the relationship of dietary inflammatory potential, as measured by energy-adjusted Dietary Inflammatory Index (E-DII) scores, with MetS and cardiometabolic risk biomarkers in an Ecuadorian group, in this case, urban adult women. It also is one of only a few to do so in a Latin American country. The study findings indicated that women with most pro-inflammatory diets had an odds for MetS that was increased nearly three-fold compared to those having diets which were the least inflammatory, i.e., anti-inflammatory, even after adjustment for potential confounders. For the most part, these relationships were dose-response since the odds of MetS increased with increasing dietary inflammation. Our study also identified positive associations between E-DII scores and levels of triglycerides and total cholesterol in fasting blood, and waist circumference. They also identified a positive association between E-DII and Framingham Risk scores suggestive of higher CVD risk.

The E-DII score range (−4.89 to 4.45) recorded for the Ecuadorian women in the sample was similar to those recorded for other Latin American studies examining the association of E-DII scores with Mets and/or cardiometabolic components in Bogota, Colombia (−3.71 to 3.64) [34], Cuernavaca and Toluca, Mexico (−4.5 to 3.8) [35], Mexico City (−5.49 to 4.12 [36] and Ribeirao Preto, Brazil (−4.69 to 5.28) [33].

Around a quarter of the Ecuadorian women in our study met the criteria for MetS. This figure is similar to the 31% reported for Ecuadorian women aged 18–59 years from a recent national survey [39]. The robust positive relationship between E-DII scores and MetS identified in our study is also consistent with two published studies of similarly aged Mexican [35] and Colombian adults [34]. Our findings on E-DII scores and cardiometabolic risk components are also partially consistent with those of prior Mexican [35,36] and Colombian studies [34]. For example, similar to our study, Canto-Osorio and associates [35] linked more pro-inflammatory diets to higher blood triglycerides and larger waist size in their cohort of ostensibly healthy Mexican adults and like our study, they also found no association with blood glucose, HDL-c. However, different from our study, they reported a lack of association between E-DII scores and total blood cholesterol.

Our findings on the association of more pro-inflammatory E-DII scores with abdominal obesity and total blood cholesterol were consistent with those of the cross-sectional study of Mexican adults published by Denova-Gutiérrez et al. [36], nearly one-sixth of whom had diagnosed T2D. However, unlike our study, they reported finding a lack of association with blood triglyceride levels and a positive association with blood glucose values.

The cross-sectional study published by Camargo-Ramos and associates [34], classified the E-DII scores of overweight and sedentary Colombian adults as either anti-inflammatory (negative values) or pro-inflammatory (positive values). Their results indicated that reported that anti-inflammatory diet was correlated with lower plasma triglyceride levels, glucose and pulse wave velocity. They also reported that pro-inflammatory diets were not associated with other blood lipid profile components, blood glucose, blood pressure, waist circumference, or BMI [34].

One possible reason for the difference in findings between our study of Ecuadorian women and the above studies is that none of them studies stratified their analyses by sex. This could account for some of differences in our findings on cardiometabolic risk indicators as these are reported to cluster by gender and reproductive hormone status (e.g., menopause) [56,57].

Age is another factor that is tightly linked to MetS and cardiometabolic risk. Our findings on the relation of E-DII scores with both MetS and individual risk factors differs from those reported for much younger Brazilian women and men, all of whom were in their early to mid-20s [33]. Specifically, the authors of this sex-stratified study failed to identify any associations between E-DII scores and MetS or cardiometabolic risk factors they measured in women including abdominal obesity and blood triglycerides. The reason for this difference is unclear but may be because cardiometabolic risk biomarkers are less evident and cluster differently in younger persons [58]. Thus, any adverse effects caused by the habitual intake of more pro-inflammatory foods may have not had sufficient time to have become manifest. It is also possible that the low prevalence of MetS (12.2%) recorded for their sample could have reduced the amount of statistical contrast available for the analyses.

Our findings on the association of E-DII scores with MetS and/or cardiometabolic risk components in Ecuadorian women are consistent with some but not studies that reported on women or stratified their results by sex in non-Latin American populations. For example, our findings on the relationship of E-DII scores with MetS concur with those published on women participants in a South Korean study [29] but differ from the lack of association reported for other South Korean [32] or Chinese women [30]. They also are in agreement with those studies identifying a positive association between E-DII scores and waist size [28,29] or fasting blood triglycerides [29] in women from Spain and/or South Korea. However, they disagree with the results published on E-DII scores and waist measurements [30,32], BMI [28], blood triglyceride levels [30,32], blood HDL-c and blood glucose levels [29,30,32] in women from countries including Spain, China, Korea and Iran.

The reasons for the differences in findings reported by our study and that of other authors inside and outside of Latin America are not immediately evident. It is likely some of the discrepancies may be the result of methodological differences including those related to study design, sample size and power, statistical analysis, biomarker measurement methods, variation in the number of E-DII food parameters used, and those related to dietary assessment such as the instrument used (e.g., FFQ, 24-dietary recall), the specific foods, and length of time covered by those assessments. For example, some of the anti-inflammatory items such turmeric, saffron, ginger and rosemary that were referenced in the original E-DII are never or very rarely consumed in Ecuador especially among low-income women such as the study participants.

Population differences could also be responsible for the between-study differences. In addition to the previously noted factors such as age [2,59] sex/hormonal profile [53,54], other non-modifiable and modifiable characteristics [59,60] may promote or protect against oxidative stress, chronic low-grade inflammation, cardiometabolic risk. Examples include physical activity [61,62], smoking [63,64], poor sleep [65], chronic stress [2,59,60], autoimmune diseases and infections [2,59] and chronic exposure to indoor and outdoor air pollutants [2,66,67]. In addition, unmeasured exposures to pro-inflammatory inorganic and organic environmental contaminants such as toxic metals/metalloids (lead, arsenic, mercury), mycotoxins, pesticides/fertilizers, and per- or poly-fluoroalkyl substances (PFAS) that are present in foods and spices, the water used for food preparation, or those generated during cooking (e.g., polycyclic aromatic hydrocarbons) could have impacted the findings among studies. These contaminants have well-documented effects on systemic inflammation, cardiometabolic risk factors, and cardiometabolic disease [68,69,70].

Our population-based study had strengths and limitations. One its strengths was that the anthropometric, laboratory, and clinical cardiometabolic biomarkers were collected from participants by well-trained investigators following standard protocols. This approach reduces measurement error, memory bias, and other potential sources of error that often occurs in studies using self-reported data. In addition, the semi-quantitative FFQ used in the study was population-specific, developed for women living in low-income neighborhoods in Quito, and it contained a wide variety of 133 commonly eaten foods, beverages, and spices. We also used the comprehensive USDA food composition database to analyze the nutrients, flavonoids, and other food components from the FFQ for the E-DII analysis. Furthermore, we adjusted for potential confounders and effect modifiers using multivariate linear and logistic regression models.

One of the limitations of our study was its cross-sectional design which does not allow for the establishment of causal or temporal relationships. Also, as non-probabilistic sampling was used to select participants, it may not be representative of women living in other areas of Quito. As with any type of self-reported dietary assessment method, measurement error caused by recall bias, social bias, or other types of bias could have occurred. In addition, since the FFQ covered only the prior 12-months women’s longer-term intake of specific foods could have been under-or over-estimated. In addition, the E-DII scores in this work were based on 29 nutritional parameters rather than the full 45 contained in the original E-DII [19]. This occurred because some of items were very rarely or never consumed by the low-income Quito populations. However, other authors have reported that such a reduction in the number of nutritional parameters does not appear to compromise the E-DII’s discriminatory potential [71]. Some of the analyses examining the association of the E-DII with some of the cardiometabolic biomarkers could have been underpowered due to the relatively small sample size of 276 women. Finally, although our multivariable analyses adjusted for participant age, age, ethnicity, education, household *per capita* income, and/or BMI, it is possible that, confounding could have occurred due to unmeasured factors.

In conclusion, our study findings suggest that the consumption of more pro-inflammatory diets appears to promote poorer cardiometabolic health as indicated by MetS, individual cardiometabolic risk components, and Framingham Risk Scores. Prospective studies should be undertaken to confirm the present study findings and elucidate the role of diet in promoting chronic low-grade inflammation and cardiometabolic risk in pre-menopausal women including those living in populations undergoing nutritional and epidemiologic transition. These can help to inform public health policy and the design of clinical interventions featuring diets with lower inflammatory potentials that can prevent or decelerate development of cardiometabolic disorders in women.

## Figures and Tables

**Table 1:** Individual, household, and neighborhood characteristics of participants across quartiles of E-DII ^a^.

Characteristic	Total	Q1 −4.89 to −3.43	Q2 −3.42 to −1.63	Q3 −1.62 to 1.16	Q4 1.17 to 4.45	*p* ^b^
No. of participants	276	69	69	69	69	
Age (years)	35.5 ± 8.1	35.3 ± 8.7	35.3 ± 8.5	35.3 ± 6.3	36.1 ± 8.7	0.91
Self-reported ethnicity						
Mestizo majority	92.4	91.3	89.9	94.2	94.2	0.50
Indigenous or afro-descendant minority	7.6	8.7	10.1	5.8	5.8	
Education (years)	7.7 ± 3.7	8.2 ± 3.8	7.7 ± 3.8	7.1 ± 3.3	7.9 ± 3.9	0.34
Marital status Legal or civil marital union	84.4	78.2	81.1	88.4	90.0	0.02
Single	6.2	2.9	14.5	5.8	1.5	
Divorced/separated/widowed	9.4	18.9	4.4	5.8	8.5	
Current employment						
Any employment outside home	55.8	50.7	59.4	53.6	59.4	0.66
Full-time homemaker	44.2	49.3	40.6	46.4	40.6	
Household size (no. of members)	4.7 ± 1.4	4.9 ± 1.3	4.5 ± 1.6	4.7 ± 1.3	4.8 ± 1.5	0.53
*Per capita* income (US$/month)	125.4 ± 88.9	121.5 ± 107.2	140.1 ± 104.1	121.6 ± 75.3	118.3.6 ± 60.4	0.46
Neighborhood of residence						
Cotocollao	41.3	45.6	40.6	40.6	38.2	0.28
El Camal	35.9	29.4	31.9	33.3	47.1	
Los Chillos	22.8	25.0	27.5	26.1	14.7	
SBP (mmHg)	115.5 ± 13.5	111.9 ± 12.1	116.5 ± 14.8	116.6 ± 13.1	117.1 ± 13.4	0.08
DBP (mmHg)	66.8 ± 10.5	63.0 ± 8.3	66.4 ± 11.4	69.2 ± 10.1	68.4 ± 11.2	0.002
Total cholesterol (mg/dL)	179.3 ± 34.9	159.6 ± 31.7	179.1 ± 28.8	180.5 ± 31.7	198.0 ± 36.6	<0.001
Triglycerides (mg/dL)	145.9 ± 88.4	87.7 ± 44.3	116.9 ± 28.9	166.4 ± 83.0	212.4 ± 113.8	<0.001
HDL-c (mg/dL)	48.5 ± 14.8	50.6 ± 14.5	48.8 ± 14.1	46.3 ± 11.9	48.4 ± 18.0	0.40
LDL-c (mg/dL)	103.3 ± 29.2	92.7 ± 28.4	106.2 ± 27.3	103.9 ± 28.8	109.9 ± 29.9	0.005
Blood glucose (mg/dL)	87.2 ± 17.8	87.4 ± 19.8	84.0 ± 9.3	85.5 ± 9.5	90.9 ± 26.3	0.16
Waist circumference (cm)	88.2 ± 14.3	82.4 ± 18.5	87.8 ± 13.2	90.3 ± 9.6	92.1 ± 12.7	<0.001

Abbreviations: DII, dietary inflammatory index; Q, quartile. ^a^ Data are means ± SDs or proportions. ^b^ *p* values were calculated for any difference across quartiles of DII, using students’ independent *t*-test or ANOVA (continuous variables) or X^2^ test (categorical variables). The clinical, lab, and anthropometric summary statistics are non-log transformed.

**Table 2:** Dietary energy and nutrient intake across E-DII quartiles of Ecuadorian women participants (*n* = 276) ^a^.

Energy & Nutrients ^b^	Q1 −4.89 to −3.43	Q2 −3.42 to −1.63	Q3 −1.62 to 1.16	Q4 1.17 to 4.45	*p* ^c^
Energy (kcal)	1788.4	2136.9	2302.4	2477.7	<0.001
Carbohydrate (g)	286.7	287.4	292.3	360.1	<0.001
Protein (g)	122.8	102.9	99.6	82.7	<0.001
Total fat (g)	102.6	126.0	118.3	157.6	<0.001
MUFA (g)	14.2	19.5	25.1	32.7	<0.001
PUFA (g)	21.3	17.6	16.6	17.0	0.051
n-3 fatty acids (g)	0.6	0.5	0.5	0.4	<0.001
n-6 fatty acids (g)	5.8	5.8	4.1	3.2	<0.001
Saturated fat (g)	45.2	42.1	33.9	32.9	<0.001
*Trans* fat (g)	0.4	0.4	0.3	0.2	<0.001
Cholesterol (mg)	253.7	210.4	170.7	184.6	<0.001
Fiber (g)	29.3	24.0	25.9	20.8	<0.001
Vitamin A (RE)	1473.1	1037.0	1377.6	885.7	<0.001
Vitamin B_6_ (mg)	3.39	2.87	2.2	1.6	<0.001
Vitamin B_12_ (mg)	5.90	5.01	4.2	2.7	<0.001
Vitamin C (mg)	121.4	124.7	92.1	110.3	0.141
Vitamin D (µg)	10.0	7.2	5.8	3.8	<0.001
Vitamin E (mg)	31.3	26.8	20.4	12.9	<0.001
Niacin (mg)	27.1	23.4	18.2	23.6	0.030
Magnesium (mg)	497.6	491.9	362.7	349.7	<0.001
Iron (mg)	25.8	20.9	16.7	12.3	<0.001
Zinc (mg)	16.3	13.3	11.4	7.7	<0.001
Riboflavin	3.0	2.6	2.0	1.4	<0.001
Selenium	119.4	112.9	97.3	67.0	<0.001
Thiamin	2.7	2.4	1.9	1.5	<0.001
Folic acid	292.9	209.6	195.9	156.5	<0.001
Beta carotene	4690.3	3441.1	3045	2509.2	<0.001
Alcohol	2.0	0.4	0.5	0.1	0.233
Caffeine	13.2	12.1	10.3	6.5	0.108
Flavan-3-ol	48.8	44.1	28.9	19.1	<0.001
Flavones	4.1	3.0	2.0	1.3	<0.001
Flavanols	27.2	18.1	16.6	14.5	<0.001
Flavanones	23.6	18.2	15.8	9.5	<0.001
Isoflavones	13.5	12.7	9.9	7.8	<0.001
Anthocyanins	38.6	36.6	26.6	20.0	<0.001
Black tea	0.1	0.3	0.2	0.4	0.610
Garlic	8.0	5.1	3.9	2.5	<0.001
Onion	68.9	56.4	53.6	32.9	<0.001
Pepper	0.2	0.1	0.1	0.1	0.038

Abbreviations: DII, dietary inflammatory index; No., number; Q, quartile. ^a^ Data are means. ^b^ All nutrients were energy-adjusted. ^c^ *p* values were calculated for any difference across quartiles of DII, using ANOVA.

**Table 3:** The associations [ORs (95% CIs)] between E-DII score quartiles and risk of metabolic syndrome (MetS) among 276 participants ^a^.

	Q1 −4.89 to −3.43	Q2 −3.42 to −1.63	Q3 −1.62 to 1.16	Q4 1.17 to 4.45	Linear Association	
					DII (Per 1-Unit Increment)	*p* ^b^
Model 1 ^c^	Ref.	0.51 (0.20, 1.32)	3.40 (1.60, 7.22)	4.54 (2.14, 9.45)	1.36 (1.21, 1.51)	<0.001
Model 2 ^d^	Ref.	0.49 (0.19, 1.29)	3.45 (1.58, 7.50)	4.38 (1.99, 9.63)	1.36 (1.22, 1.52)	<0.001

Abbreviations: CI: confidence interval; DII-e: dietary inflammatory index; OR: odds ratio; Q: quartile. ^a^ All models were constructed using logistic regression model. ^b^ *p* values for linear trend were calculated using the exposure as the continuous variable. ^c^ Model 1 was the crude model. ^d^ Model 2 was adjusted for age, ethnicity, education, and household *per capita* income.

**Table 4:** The associations (beta regression coefficients (95% CIs)) between E-DII score quartiles and normalized cardiometabolic risk indicators among the 276 women participants ^a^.

	Q1 −4.89 to −3.43	Q2 −3.42 to −1.63	Q3 −1.62 to 1.16	Q4 1.17 to 4.45	*p* for Linear Trend ^b^
Total cholesterol					
Model 1 ^c^	Ref.	0.052 (0.025, 0.080)	0.055 (0.028, 0.082)	0.094 (0.067, 0.122)	<0.001
Model 2 ^d^	Ref.	0.053 (0.025, 0.080)	0.054 (0.027, 0.082)	0.095 (0.066, 0.123)	<0.001
HDL-c					
Model 1 ^c^	Ref.	−0.017 (−0.061, 0.028)	−0.036 (−0.080, 0.009)	−0.028 (−0.073, 0.017)	0.421
Model 2 ^d^	Ref.	−0.015 (−0.060, 0.030)	−0.034 (−0.079, 0.011)	−0.024 (−0.070, 0.022)	0.511
LDL-c					
Model 1 ^c^	Ref.	0.063 (0.018, 0.108)	0.051 (0.006, 0.096)	0.076 (0.031, 0.121)	0.006
Model 2 ^d^	Ref.	0.064 (0.018, 0.109)	0.050 (0.004, 0.095)	0.077 (0.030, 0.124)	0.007
Waist circumference					
Model 1 ^c^	Ref.	0.036 (0.006, 0.067)	0.052 (0.022, 0.082)	0.057 (0.027, 0.088)	<0.001
Model 2 ^e^	Ref.	0.032 (0.004, 0.060)	0.045 (0.017, 0.074)	0.040 (0.012, 0.069)	0.008
Body mass index					
Model 1 ^c^	Ref.	0.007 (−0.019, 0.032)	0.020 (−0.005, 0.046)	0.042 (0.017, 0.068)	0.007
Model 2 ^e^	Ref.	0.002 (−0.001, 0.006)	0.003 (−0.001, 0.007)	−0.001 (−0.004, 0.003)	0.151
Glucose					
Model 1 ^c^	Ref.	−0.012 (−0.035, 0.011)	0.001 (−0.022, 0.023)	0.014 (−0.009, 0.036)	0.169
Model 2 ^d^	Ref.	−0.012 (−0.035, 0.010)	0.002 (−0.024, 0.021)	0.008 (−0.015, 0.031)	0.380
Systolic blood pressure					
Model 1 ^c^	Ref.	0.017 (0.001, 0.033)	0.018 (0.002, 0.034)	0.019 (0.003, 0.035)	0.064
Model 2 ^d^	Ref.	0.016 (0.001, 0.031)	0.015 (−0.001, 0.030)	0.012 (−0.003, 0.029)	0.137
Diastolic blood pressure					
Model 1 ^c^	Ref.	0.021 (−0.001, 0.043)	0.041 (0.019, 0.062)	0.035 (0.013, 0.056)	0.001
Mean arterial pressure					
Model 1 ^c^	Ref.	0.019 (0.002, 0.036)	0.030 (0.013, 0.047)	0.028 (0.010, 0.045)	0.003
Model 2 ^d^	Ref.	0.019 (0.003, 0.036)	0.029 (0.012, 0.045)	0.023 (0.007, 0.040)	0.006

Abbreviations: CI: confidence interval; DII: dietary inflammatory index; Q: quartile. ^a^ All models were constructed using linear regression; ^b^ *p* values for linear trend were calculated using the exposure as the continuous variable.^c^ Model 1 was the unadjusted model; ^d^ Model 2 was adjusted for age, ethnicity, education, household per capita income, and BMI; ^e^ Model 2 was adjusted for age, ethnicity, education, and household per capita income.

**Table 5:** The associations between E-DII score quartiles and Framingham risk score (FRS) among the 276 women participants ^a^.

	Q1 −4.89 to −3.43	Q2 −3.42 to −1.63	Q3 −1.62 to 1.16	Q4 1.17 to 4.45	*p* for Linear Trend ^b^
FRS	−1.6 ± 5.0	0.6 ± 4.6	0.2 ± 3.7	2.0 ± 4.6	<0.001

Abbreviations: DII: dietary inflammatory index; Q: quartile. ^a^ Data are means ± SDs; ^b^ *p* values for linear trend were calculated using linear regression model.

## Data Availability

The data that support the findings of this study are available from the corresponding author, (MW), upon reasonable request.
